# Effects of a Dulaglutide plus Calorie-Restricted Diet versus a Calorie-Restricted Diet on Visceral Fat and Metabolic Profiles in Women with Polycystic Ovary Syndrome: A Randomized Controlled Trial

**DOI:** 10.3390/nu15030556

**Published:** 2023-01-20

**Authors:** Yuqin Zhang, Zhihua Qu, Ting Lu, Xiaowen Shao, Meili Cai, Diliqingna Dilimulati, Xinxin Gao, Weiqing Mao, Fan Hu, Lili Su, Qiong Liao, Ting Han, Manna Zhang, Shen Qu

**Affiliations:** 1 Department of Endocrinology and Metabolism, Shanghai Tenth People’s Hospital, School of Medicine, Tongji University, Shanghai 200072, China; 2 Department of Nutrition, Shanghai Tenth People’s Hospital, School of Medicine, Tongji University, Shanghai 200072, China; 3 Department of Obstetrics and Gynecology, Shanghai Tenth People’s Hospital, School of Medicine, Tongji University, Shanghai 200072, China; 4 Department of Nuclear Medicine, Shanghai Tenth People’s Hospital, School of Medicine, Tongji University, Shanghai 200072, China

**Keywords:** polycystic ovary syndrome, dulaglutide, calorie-restricted diet, visceral adipose tissue, glucagon-like peptide 1 receptor agonist

## Abstract

The effects of dulaglutide and a calorie-restricted diet (CRD) on visceral adipose tissue (VAT) and metabolic profiles in polycystic ovary syndrome (PCOS) have not been extensively investigated. In this study, we investigated whether dulaglutide combined with CRD could further reduce VAT and promote clinical benefits as compared with a CRD regimen alone in overweight or obese PCOS-affected women. Between May 2021 and May 2022, this single-center, randomized, controlled, open-label clinical trial was conducted. Overall, 243 participants with PCOS were screened, of which 68 overweight or obese individuals were randomly randomized to undergo dulaglutide combined with CRD treatment (*n* = 35) or CRD treatment alone (*n* = 33). The duration of intervention was set as the time taken to achieve a 7% weight loss goal from baseline body weight, which was restricted to 6 months. The primary endpoint was the difference in the change in VAT area reduction between the groups. The secondary endpoints contained changes in menstrual frequency, metabolic profiles, hormonal parameters, liver fat, and body composition. As compared with the CRD group, the dulaglutide + CRD group had a considerably shorter median time to achieve 7% weight loss. There was no significant between-group difference in area change of VAT reduction (−0.97 cm^2^, 95% confidence interval from −14.36 to 12.42, *p* = 0.884). As compared with CRD alone, dulaglutide + CRD had significant advantages in reducing glycated hemoglobin A1c and postprandial plasma glucose levels. The results of the analyses showed different changes in menstruation frequency, additional metabolic profiles, hormonal markers, liver fat, and body composition between the two groups did not differ significantly. Nausea, vomiting, constipation, and loss of appetite were the main adverse events of dulaglutide. These results emphasize the value of dietary intervention as the first line of treatment for PCOS-affected women, while glucagon-like peptide 1 receptor agonist therapy provides an efficient and typically well tolerated adjuvant therapy to aid in reaching weight targets based on dietary therapy in the population of overweight/obese PCOS-affected women.

## 1. Introduction

Polycystic ovary syndrome (PCOS) is a unification of reproductive endocrine and metabolic disorders that affects 6–20% of reproductive-aged women [1]. Approximately 60–70% of women with PCOS are overweight or obese [2]. The increased abdominal phenotype of fat distribution is a common manifestation in both normal-weight and overweight/obese women with PCOS, which forms vicious circles with hyperandrogenic states and insulin resistance (IR) [3,4]. Visceral adipose tissue (VAT) can secrete more metabolically harmful adipokines and inflammatory factors as compared with subcutaneous adipose tissue (SAT), which can aggravate IR and can also worsen oligo/anovulation and hyperandrogenism [5,6]. Therefore, it is important to reduce visceral adiposity in the PCOS population, particularly women who are overweight or obese.

Lifestyle and weight management, particularly dietary intake aimed at weight loss, are initial treatment strategies for PCOS. Weight loss of 5–10% from baseline body weight ameliorates insulin sensitivity and the ovulation rate, and decreases hyperandrogenism among the overweight/obese PCOS population [7,8]. A calorie-restricted diet (CRD) seems to be the optimal dietary pattern for weight management in the PCOS population, which could reduce body weight, improve metabolic and reproductive parameters, and increase the probability of conception [9,10,11]. However, there are limited data on how the CRD regimen acts on visceral adiposity in PCOS-affected women.

Glucagon-like peptide 1 (GLP-1) is a peptide hormone released by gut enteroendocrine cells that improves glucose homeostasis and reduces body weight [12,13]. Recently, GLP-1 analogs with receptor agonists (RAs) have been studied for treating PCOS, and have shown considerable improvements in insulin sensitivity, reproductive function, weight reduction, and reduced VAT in the PCOS population [14,15,16]. Dulaglutide is a once-weekly GLP-1 RA with higher adherence than other GLP-1 RAs that require daily injections [17]. However, it is unclear whether once-weekly GLP-1 RAs combined with the CRD regimen is more beneficial in reducing VAT and improving metabolic risk factors than the CRD regimen alone. Therefore, we conducted a randomized clinical trial to evaluate modifications in fat distribution, the androgenic state, and metabolic profiles in the overweight and obese PCOS-affected population, who obtained modest and equivalent weight loss induced by a CRD regimen with or without dulaglutide.

## 2. Materials and Methods

### 2.1. Trial Design and Ethics Statements

This trial was a randomized, controlled, open-label study conducted in Shanghai Tenth People’s Hospital between May 2021 and May 2022. The study protocol was approved by the Ethics Committee of Shanghai Tenth People’s Hospital (approval number SHSY-IEC-4.1/21-119/01), and the clinical trial registration number was NCT04876027. Written informed consent was acquired by each subject.

### 2.2. Eligibility Criteria

Participants were eligible if they were 18–45 years, had a body mass index (BMI) ≥24 kg/m^2^, and were diagnosed with PCOS according to the 2003 Rotterdam diagnostic criteria [18]. Exclusion criteria were any contraindication to dulaglutide (hypersensitivity to the active ingredients or any excipients of dulaglutide, multiple endocrine neoplasia syndrome type, personal or family history of medullary thyroid carcinoma); treatment with any additional medications that might impede the trial, including GLP-1 RAs, metformin, pioglitazone, contraceptives, or traditional Chinese medicine within the past 3 months; pregnancy or lactation; mental illness; malignant tumors; chronic kidney disease or severe liver dysfunction; inflammatory bowel disease; and involvement in other weight-loss research programs within the past 3 months.

### 2.3. Randomization and Intervention Programs

After a baseline evaluation, participants were randomized to receive dulaglutide combined with CRD or CRD alone, hinged on the predetermined number generated by a computer with a 1:1 allocation, which was concealed using opaque, sealed, and serially numbered envelopes. Participants remained on their prescribed treatment program until they reached the 7% weight loss goal (determined by initial body weight at randomization), which was reported to be associated with ameliorative metabolic outcomes [19,20]. The groups both received dietary counseling and were urged to engage in more physical activities throughout the program (30 min of moderate-intensity aerobic exercise per day, 5–7 days a week, and 10–20 min of resistance exercise, 3 times a week). Participants in the dulaglutide + CRD group received dulaglutide treatment weekly at a dose of 1.5 mg by subcutaneous injection (Trulicity, Eli Lilly). Participants were advised to avoid pregnancy during dulaglutide injection. To be eligible for the project, participants were encouraged to reach a 7% weight loss goal within 6 months after being randomized. The percentage of treatment compliance was determined by the number of participants who reached the target weight loss within 6 months among the participants recruited in each group.

Dietary consultation was conducted by dietitians from the Department of Nutrition in the hospital. Once enrolled in the trial, participants received personalized dietary information booklets that provided portion advice and specific one-week recipes. CRD is a dietary pattern that maintains basic nutritional requirements while restricting energy intake, with a ratio of macronutrients meeting the requirements of a balanced diet. Dietitians calculated daily calorie requirements using the following formula: (height (cm) – 105) × 20 kcal, and all participants were instructed to follow a diet of 1000 to 1300 kcal per day. In the diet, carbohydrates accounted for 40–55% of the total calories, fat 20–30%, and protein 15–20% [21], which was individualized according to the participants’ food preferences, intolerances, and optimal portion sizes for each food group in the Food Guide Pyramid. Dietitians explained, in detail, food exchange to the participants in order to increase dietary diversity. During the trial period, participants were advised to weigh food at the beginning to ascertain approximately how much food met their caloric intake per meal. All participants were required to record their diet and physical activity in a daily dietary log, photograph the food they ate, upload photographs to WeChat (an application for online communication), and report their weight at least twice a week. On WeChat, dietitians assessed the participants’ dietary intakes each day and provided advice to ensure timely dietary adjustments. Participants met with dietitians every month in the hospital to assess their compliance with the prescribed dietary intervention, bolster their determination to lose the desired amount of weight, and evaluate adverse events. Diet protocol violation was considered a failure to adhere to the dietary program, which was defined as the percentage of adherence being <80%, even after being well instructed by the dietitians; this was specifically reflected in uploading photographs of substandard diets and not photographing or recording diets so that the researchers were unaware of what they ate. The percentage of adherence to the prescribed diets was determined by the number of days that each participant met the dietary requirements during the intervention period.

### 2.4. Trial Outcomes

The primary outcome was the between-group difference in the change from baseline in the VAT area at the time of achieving 7% weight loss goal. The secondary outcomes included changes in menstrual frequency, blood pressure, metabolic risk factors, reproductive hormones, liver fat content, percentage of total body fat, percentage of total body lean, total fat mass, total lean mass, and abdominal SAT mass, which were evaluated after the accomplishment of the 7% weight loss goal. Body weight and waist circumference (WC) were also recorded. The BMI was calculated as follows: body weight (kg)/height (m^2^). The systolic blood pressure (SBP) and diastolic blood pressure (DBP) were measured. Menstrual frequency was defined as the number of menstruations over the past 12 months. Metabolic risk factors were measured including fasting plasma glucose (FPG), fasting insulin (FINS), glycated hemoglobin A1c (HbA1c), alanine aminotransferase (ALT), aspartate aminotransferase (AST), total cholesterol (TC), triglycerides (TG), high-density lipoprotein cholesterol (HDL-c), low-density lipoprotein cholesterol (LDL-c), creatinine, serum uric acid (SUA), and sex hormone-binding globulin (SHBG). Postprandial plasma glucose (PPG) and postprandial insulin levels were measured at 120 min using a 75 g oral glucose tolerance test (OGTT). Reproductive hormones included the luteinizing hormone (LH), follicle-stimulating hormone (FSH), prolactin (PRL), total testosterone (TT), free testosterone (FT), androstenedione (AD), and dehydroepiandrosterone sulfate (DHEAS). The free androgen index (FAI) was calculated as TT (nmol/L)/SHBG (nmol/L). The homeostasis model assessment of insulin resistance (HOMA–IR) was calculated as (FPG (mmol/L) × FINS (mU/L))/22.5 [22]. The degree of liver fat and fibrosis was assessed using transient elastography (FibroScan 502, Echosens Inc., Paris, France) and was represented by the controlled attenuation parameter (CAP) and liver stiffness measurement (LSM), respectively. Whole-body composition and fat distribution assessed by dual-energy X-ray absorptiometry (DXA) (APEX 4.5.0.2; Hologic Inc., Marlborough, Massachusetts, USA) were performed at baseline and after prespecified weight loss [23,24]. The DXA fat depot measurements in the abdomen were located above the iliac crest, at a level approximately parallel to the fourth lumbar vertebra, and within an area 5 cm wide. The software automatically identifies the inner and outer edges of the abdominal walls on both sides of the projected DXA image according to the profile of total abdominal fat and lean mass flush with the fourth lumbar vertebra. Then, the software performs measurements of total abdominal fat mass (TAT) within the abdominal walls. The abdominal amount of SAT was measured between the skin line and outer abdominal wall on both sides of the DXA image. DXA-VAT was calculated by subtracting SAT from TAT within the visceral region [25]. The percentage of total body fat, percentage of total body lean, total fat mass, and total lean mass were automatically calculated using the DXA software.

### 2.5. Sample Size Estimation and Statistical Analysis

Based on a previous study [26], with a two-tailed alpha of 0.05, power of 90%, and mean difference in the VAT area of at least 6.5 cm^2^ (±6.4) between dulaglutide + CRD and CRD alone, 22 participants were required for each treatment group. Considering a 30% dropout rate or lack of achievement of prespecified weight loss, 32 participants were included in each treatment group.

The intention-to-treat principle was applied to all data analysis. Baseline data are presented as means with 95% confidence intervals (CIs) for continuous variables. The median time to event and associated 95% CIs were calculated after the Kaplan–Meier method was applied to analyze time-to-event data. Hazard ratios (HRs) with 95% CIs were calculated using Cox proportional hazards models after adjusting for age and the BMI. Mean differences between the two treatment groups are presented as least-squares mean changes with 95% CIs. Group differences in the primary and secondary outcomes were evaluated using covariance analysis for continuous variables, with the corresponding baseline assessment as the covariate and randomized treatment as the fixed factor, and the chi-square test or Fisher exact test for categorical variables where appropriate. A two-sided *p*-value <0.05 was considered to be statistically significant. All statistical analyses were performed using SPSS, version 25.0 (SPSS Inc., Chicago, IL, USA).

## 3. Results

### 3.1. Trial Participants

**Figure 1:**
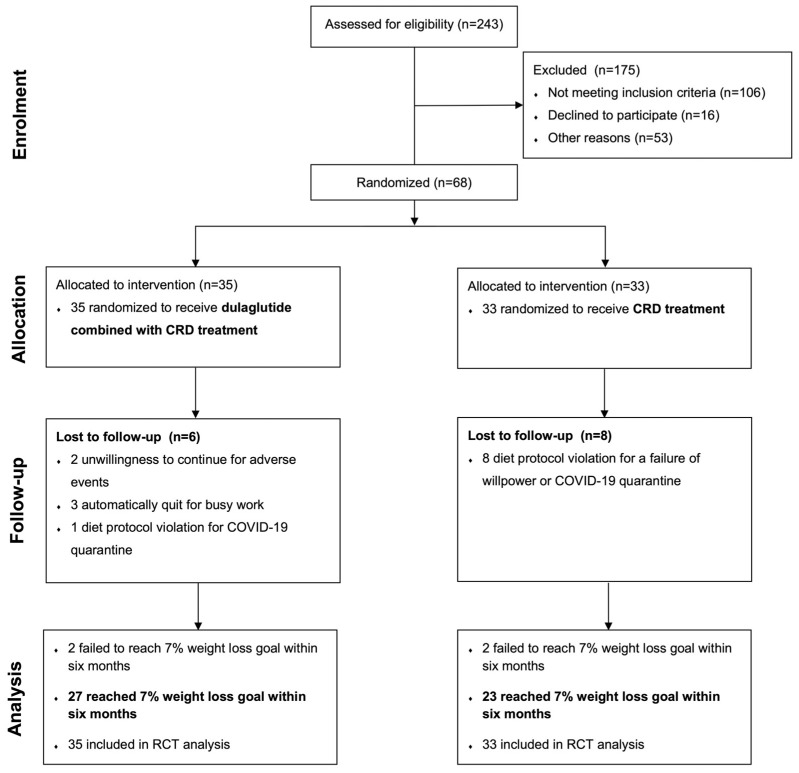
Flow diagram of this randomized, controlled, open-label trial. CRD, calorie-restricted diet. RCT, randomized controlled trial.

From May 2021 to May 2022, 243 women were assessed for eligibility. Among them, 175 were excluded considering inclusion/exclusion criteria or other reasons (106 women did not match the criteria for inclusion, 16 women declined to participate, and 53 women were excluded for other reasons). A total of 68 overweight or obese PCOS-affected participants were randomly assigned to the following groups: 35 participants to the dulaglutide + CRD group and 33 participants to the CRD group. Among the total number of women who underwent randomization, 27 women in the dulaglutide + CRD group and 23 women in the CRD group completed a 7% weight loss goal of initial body weight within 6 months. Additionally, 4 women failed to obtain the predefined weight loss within the allowed 6-month period and 14 women were lost to follow-up (2 women for unwillingness to continue for adverse events, 3 women for automatically quitting, and 9 women for diet protocol violation) (Figure 1).

### 3.2. Baseline Characteristics and Adherence

At baseline, clinical characteristics of subjects were similar between two the groups (Table 1). Subjects in the dulaglutide + CRD group had a BMI of 29.68 kg/m^2^, mean TT level of 1.76 nmol/L, and mean VAT area of 163.03 cm^2^. Subjects in the CRD group had a BMI of 29.71 kg/m^2^, mean TT level of 1.84 nmol/L, and mean VAT area of 156.74 cm^2^ (Table 1). The mean (95% CI) percentages of adherence to the prescribed diet in participants who reached the target weight loss within 6 months were 92.72% (95% CI from 90.31 to 95.13) in the dulaglutide + CRD group and 93.09% (95% CI from 90.70 to 95.48) in the CRD group, and there was no significant between-group difference (Supplementary Figure S1).

### 3.3. Weight Loss

**Figure 2:**
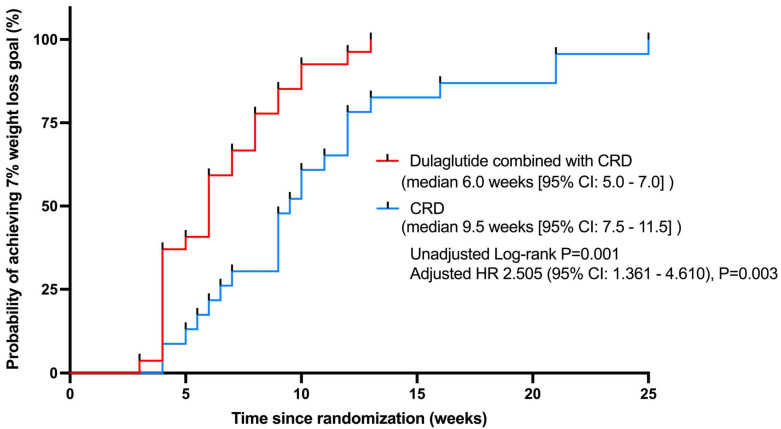
Time-to-event analysis of achieving 7% weight loss of initial body weight in the two groups. CRD, calorie-restricted diet; HR, hazard ratio.

According to the prespecified protocol, participants in both groups lost the same amount of weight. In terms of the absolute amount, the mean weight losses were −5.42 kg (95% CI from −5.79 to −5.05) in the dulaglutide + CRD group and −5.44 kg (95% CI from −5.84 to −5.04) in the CRD group (Table 2). The median time required to reach the predetermined weight loss goal was different between the groups (dulaglutide + CRD group, 6.0 weeks (95% CI from 5.0 to 7.0) and CRD group, 9.5 weeks (95% CI from 7.5 to 11.5) in log-rank *p* = 0.001). Treatment modality was an independent factor that affected the time to attainment of the 7% weight loss goal when adjusting for age and the BMI (*p* = 0.003). Participants in the dulaglutide + CRD group took a shorter time to achieve comparable weight loss as compared with those in the CRD group (HR = 2.505, 95% CI from 1.361 to 4.610) (Figure 2).

### 3.4. Body Composition and Fat Distribution

**Figure 3:**
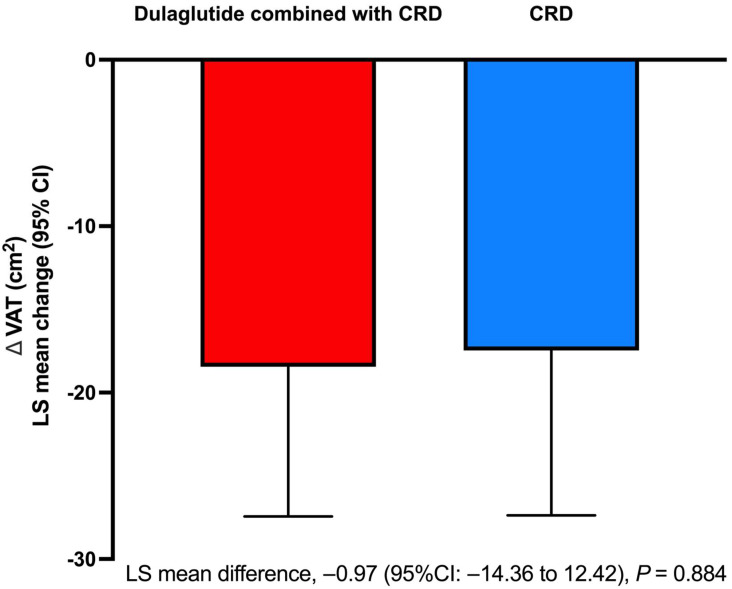
The change in VAT area from baseline to reach 7% weight loss of initial body weight in the intention-to-treat population. CRD, calorie-restricted diet; LS, least-squares; VAT, visceral adipose tissue.

After comparable loss in body weight, VAT area at the time of accomplishing the weight loss target was comparably reduced by 18.44 cm^2^ (95% CI from −27.44 to −9.43) from baseline in the dulaglutide + CRD group and by 17.47 cm^2^ (95% CI from −27.37 to −7.56) in the CRD group. No difference was observed in VAT reduction between the groups (least-squares mean difference, −0.97 cm^2^; 95% CI from −14.36 to 12.42, *p* = 0.884) (Figure 3). With respect to body composition and fat distribution, both groups had similar decreases in total fat mass, total lean mass, and abdominal SAT mass. There were no significant changes from baseline in the percentage of total body fat and percentage of total body lean in either group at the time of achieving the 7% weight loss goal (Table 2).

### 3.5. Metabolic Risk Factors

Although there were significant improvements in glycemic control in both groups, dulaglutide + CRD provided additional benefits with regard to HbA1c and PPG levels as compared with CRD after equivalent weight loss. The groups both showed similar decreases in SBP, DBP, FINS, HOMA-IR, ALT, AST, TC, TG, and LDL-c values. The dulaglutide + CRD group appeared to have significant reductions in HDL-c levels, whereas the CRD group appeared to have significant reductions in SUA levels; however, there were no differences in the changes in HDL-c and SUA levels between the groups. With regard to liver fat content, a significant decrease in CAP was observed in the CRD group, whereas a significant decrease was observed in LSM in the dulaglutide + CRD group; yet, there were no significant differences between the groups in the changes in these parameters (Table 2).

### 3.6. Menstrual Frequency and Reproductive Hormones

Although both groups showed an improvement in menstrual frequency, no between-group difference was observed in menstrual frequency from baseline to the time of achieving a 7% weight loss goal. In terms of reproductive hormones, the CRD group showed significant decreases in TT and AD levels, and both groups showed a similar increase in FAI, but there were no significant between-group differences in the changes in these parameters after treatment. No significant changes were observed in LH, FSH, PRL, FT, DHEAS, or SHBG values in either group (Table 2).

### 3.7. Adverse Events

Neither fatalities nor severe adverse events happened during the research. The adverse event-related discontinuation rate was 2.94% (2/68) in both groups. Two participants developed gastrointestinal reactions within the first week of starting dulaglutide injection; therefore, they were unwilling to continue to dulaglutide treatment and withdrew from the trial. Approximately 37.14% of the participants with dulaglutide treatment reported at least one gastrointestinal (GI) treatment-emergent adverse event (TEAE); the most common GI TEAEs were nausea (22.86%), vomiting (20.00%), constipation (11.43%), and loss of appetite (11.43%). Generally, most GI TEAEs were mild to moderate in severity, and most were more pronounced within the first 2 weeks, and then gradually ameliorated within 1 month. Approximately 9.09% of the participants who received the CRD regimen alone reported sensations of hunger, and 3.03% of participants reported sensations of dizziness (Table 3).

## 4. Discussion

Participants in the dulaglutide + CRD group took a shorter time to achieve a 7% weight loss goal than those in the CRD group, which suggests that dulaglutide could favor high efficiency in achieving the target weight loss. This finding was generally supported by the results of previous studies that have demonstrated that GLP-1 RA was effective in weight reduction in overweight and obese women with PCOS [14,15,16,27]. It is worth noting that the participants in both groups took a shorter period to achieve 7% weight loss than those in other studies [28,29], which might be explained by the high adherence and well implemented treatment protocol in the current study.

There were no significant differences in visceral fat reduction between the groups after equivalent weight loss. The significant reduction in VAT in the CRD group was consistent with previous studies’ results that have shown that weight reduction induced by CRD was associated with a significant improvement in WC and reduced fat mass [30,31]. However, after the same degree of weight loss, dulaglutide may have no additional benefits on VAT as compared with the effect resulting from diet intervention, suggesting that the extent of weight reduction, other than the type of intervention, explained most of the favorable effects of reducing visceral fat in overweight or obese individuals with PCOS. Other research involving different populations have found similar results. Swora-Cwynar et al. reported that reductions in body weight, WC, and body fat content were comparable between 12-week low-calorie diet and isocaloric diet plus metformin groups in premenopausal obese women [30]. Schübel et al. reported no significant differences in body weight and VAT volume between intermittent calorie restriction and continuous calorie restriction in overweight or obese women [32]. Liu et al. showed that changes in body weight and area of abdominal visceral fat were not significantly different between time-restricted eating and daily calorie restriction after 12 months of intervention in obese participants [33]. Moreover, our data showed that total fat mass, total lean mass, and abdominal SAT mass were reduced simultaneously and were comparable between the groups after reaching the target weight loss, which was consistent with previous studies’ findings [33,34,35].

In this trial, we found that dulaglutide combined with CRD provided additional benefits respecting HbA1c and PPG as compared with CRD after equivalent weight loss, which was consistent with earlier clinical trial findings showing that GLP-1 RAs could result in significant improvements in HbA1c and post-OGTT glucose levels in women with PCOS [14,36]. Furthermore, we found that both groups produced similar positive effects on reducing blood pressure and levels of insulin, TC, TG, and LDL-c, which was generally in agreement with previous studies’ results [37,38,39,40,41]. Our data also showed that participants in both groups had similar reductions in aminotransferases; participants treated with CRD had reductions in CAP, whereas those treated with dulaglutide combined with CRD had reductions in LSM. Previous trials that explored the effectiveness of dulaglutide in patients with type 2 diabetes (T2D) and non-alcoholic fatty liver disease showed discordant effects on liver transaminases and liver stiffness [42,43], and CRD was considered ineffective in improving aminotransferases and liver stiffness within a short-term period [44]. Thus, further investigations are required to establish the efficaciousness of dulaglutide and CRD treatment on aminotransferase levels, liver fat content, and liver fibrosis in women with PCOS.

There was no discernible between-group difference, despite the improved menstrual frequencies from baseline to the achievement of target weight loss being comparable between the groups. This phenomenon may be explained by the fact that the short time frame in the present study was not sufficient for evaluating changes in menstrual frequency. Moreover, the CRD group showed a significant decrease in TT and AD levels. Levels of LH, FSH, PRL, FT, DHEAS, and SHBG did not alter significantly in either group after moderate reductions in body weight, which was consistent with previous studies’ findings [11,45]. The impact of CRD or dulaglutide on these reproductive hormones in PCOS-affected women requires more investigation.

Concerning adverse events, GI events are common side effects of GLP1 RAs. In our study, 37.14% of the PCOS participants with dulaglutide treatment reported at least one GI TEAE, which was comparable with those observed in individuals with T2D receiving 1.5 mg once-weekly dulaglutide as described in the Assessment of Weekly AdministRation of LY2189265 in Diabetes (AWARD) trials [46,47]. Compared with the AWARD studies [46,47], the current study had higher incidences of nausea, vomiting, constipation, and loss of appetite. A possible explanation for this might be the younger study population, and more GI events might have occurred in the higher baseline BMI subgroups [48]. The CRD group showed a low incidence of sensations of hunger and dizziness, which was comparable with findings from earlier research [49,50]. Generally, although dulaglutide has some disadvantages as compared with CRD such as the injection requirement and more adverse effects, it was well tolerated by women with PCOS, and most of the study population could adhere to the diet regimen even if they experienced GI events within the first 2 weeks.

Our study has certain limitations. First, the intervention period was comparatively short and different in the two groups, which may weaken the observed usefulness of GLP-1 RA in treating PCOS. However, long-term efficacy of dulaglutide combined with CRD remains to be studied. Second, different durations to attain the target weight loss or the implications of the trial being unblinded may have introduced bias. Third, in our experiment, physical exercise was not mandated, since our aim was to determine whether the dietary regimen in isolation or in combination with medication would act on VAT reduction. Fourth, since the endpoints of the study occurred when the targeted weight loss was reached, not all reproductive hormones were measured during menstruation. Lastly, although VAT was not measured using magnetic resonance imaging (MRI) or computed tomography (CT), DXA-VAT has been validated against either MRI or CT [51,52]. However, the current study is the first randomized controlled study to investigate the efficacy of CRD with or without dulaglutide on VAT reduction in overweight and obese women with PCOS.

## 5. Conclusions

In our study, for an identical degree of weight loss, dulaglutide combined with a CRD regimen did not produce a more pronounced reduction in visceral fat than the CRD regimen. Dulaglutide combined with CRD could favor high efficiency in achieving targeted weight loss and provide additional benefits in terms of the HbA1c and PPG levels as compared with the CRD regimen alone. The interventions both had similar positive effects in improving menstrual frequency and reducing levels of blood pressure, insulin, aminotransferases, lipids, total fat mass, total lean mass, and abdominal SAT mass after equivalent weight loss. These findings support the importance of dietary intervention as a first-line treatment in women with PCOS, and GLP-1 RA therapy offers an effective and generally tolerable adjunct therapy to aid in achieving weight targets based on dietary therapy in overweight and obese women with PCOS.

## Figures and Tables

**Table 1:** Baseline characteristics of overweight/obese patients with PCOS.

Variables	Dulaglutide Combined with CRD Therapy (*n* = 35)	CRD Therapy (*n* = 33)
Age (years)	30.31 (28.58 to 32.05)	28.64 (27.09 to 30.18)
Weight (kg)	77.62 (74.19 to 81.06)	78.84 (74.24 to 83.43)
WC (cm)	96.76 (93.61 to 99.92)	94.89 (91.45 to 98.33)
BMI (kg/m^2^)	29.68 (28.44 to 30.93)	29.71 (28.39 to 31.04)
Menstrual Cycles (no./yr)	7.44 (6.07 to 8.81)	7.90 (6.83 to 8.96)
SBP (mmHg)	124.14 (119.77 to 128.52)	127.69 (121.16 to 134.22)
DBP (mmHg)	82.06 (78.55 to 85.56)	84.38 (79.81 to 88.94)
FPG (mmol/L)	4.96 (4.77 to 5.14)	5.03 (4.85 to 5.21)
PPG (mmol/L)	7.53 (6.57 to 8.50)	7.58 (6.81 to 8.34)
FINS (mU/L)	21.54 (17.19 to 25.89)	22.59 (14.44 to 30.75)
PINS (mU/L)	139.41 (99.15 to 179.67)	149.16 (111.19 to 187.14)
HbA1c (%)	5.72 (5.61 to 5.84)	5.64 (5.48 to 5.80)
HOMA-IR	4.85 (3.79 to 5.91)	5.18 (3.26 to 7.10)
ALT (U/L)	47.99 (35.45 to 60.52)	35.06 (27.58 to 42.54)
AST (U/L)	27.91 (22.83 to 33.00)	22.08 (18.48 to 25.68)
TC (mmol/L)	4.81 (4.49 to 5.13)	4.74 (4.46 to 5.02)
TG (mmol/L)	1.64 (1.34 to 1.94)	1.64 (1.36 to 1.93)
LDL-c (mmol/L)	3.10 (2.77 to 3.44)	3.04 (2.76 to 3.32)
HDL-c (mmol/L)	1.28 (1.21 to 1.35)	1.31 (1.22 to 1.41)
Cr (umol/L)	62.06 (60.24 to 63.88)	60.94 (58.68 to 63.19)
SUA (umol/L)	396.27 (371.54 to 421.00)	372.60 (340.88 to 404.32)
LH (IU/L)	9.06 (7.34 to 10.79)	11.62 (9.73 to 13.50)
FSH (IU/L)	5.45 (4.69 to 6.20)	5.54 (5.08 to 5.99)
PRL (mIU/L)	379.84 (322.33 to 437.36)	356.00 (304.60 to 407.39)
TT (nmol/L)	1.76 (1.48 to 2.04)	1.84 (1.60 to 2.07)
FT (pg/mL)	2.53 (2.13 to 2.93)	2.40 (1.99 to 2.81)
AD (ng/mL)	4.16 (3.55 to 4.77)	4.61 (3.46 to 5.76)
DHEAS (ug/dl)	227.83 (193.64 to 262.02)	225.00 (182.71 to 267.29)
SHBG (nmol/L)	29.74 (16.80 to 42.67)	23.96 (14.59 to 33.33)
FAI	0.09 (0.07 to 0.11)	0.11 (0.08 to 0.13)
CAP (dB/m)	332.69 (314.13 to 351.25)	318.70 (300.50 to 336.89)
LSM (kPa)	5.95 (5.29 to 6.61)	5.82 (5.00 to 6.64)
Total body fat (%)	43.27 (42.08 to 44.46)	43.57 (42.25 to 44.89)
Total body lean (%)	53.50 (52.37 to 54.64)	52.75 (51.44 to 54.07)
Total fat mass (kg)	33.03 (30.94 to 35.11)	33.50 (31.20 to 35.80)
Total lean mass (kg)	40.68 (39.13 to 42.23)	40.29 (37.85 to 42.73)
VAT area (cm^2^)	163.03 (146.19 to 179.88)	156.74 (140.42 to 173.05)
SAT mass (kg)	1.96 (1.79 to 2.13)	1.92 (1.73 to 2.12)

The data are presented as the mean (95% CI). AD, androstenedione; ALT, alanine aminotransferase; AST, aspartate aminotransferase; BMI, body mass index; CAP, controlled attenuation parameter; Cr, Creatinine; CRD, calorie-restricted diet; DBP, diastolic blood pressure; DHEAS, dehydroepiandrosterone sulfate; FAI, free androgen index; FINS, fasting insulin; FPG, fasting plasma glucose; FSH, follicle-stimulating hormone; FT, free testosterone; HbA1c, glycosylated hemoglobin A1c; HDL-c, high- density lipoprotein cholesterol; HOMA-IR, homeostasis model assessment of insulin resistance; LDL-c, low-density lipoprotein cholesterol; LH, luteinizing hormone; LSM, liver stiffness measurement; PCOS, polycystic ovary syndrome; PINS, postprandial insulin; PPG, postprandial plasma glucose; PRL, pituitary prolactin; SAT, subcutaneous adipose tissue; SBP, systolic blood pressure; SHBG, sex hormone-binding globulin; SUA, serum uric acid; TC, total cholesterol; TG, triglyceride; TT, total testosterone; VAT, visceral adipose tissue; WC, waist circumference.

**Table 2:** Secondary efficacy endpoints.

Variables	Dulaglutide Combined with CRD Therapy, LS Mean Change (95% CI) (*n* = 35)	CRD Therapy, LS Mean Change (95% CI) (*n* = 33)	LS Mean Difference between Groups (95% CI)	*p*
Weight (kg)	−5.42 (−5.79 to −5.05)	−5.44 (−5.84 to −5.04)	0.02 (−0.53 to 0.57)	0.945
WC (cm)	−4.34 (−5.55 to −3.14)	−3.61 (−5.05 to −2.17)	−0.73 (−2.61 to 1.15)	0.435
BMI (kg/m^2^)	−2.06 (−2.25 to −1.87)	−2.23 (−2.43 to −2.02)	0.17 (−0.11 to 0.45)	0.232
Menstrual Cycles (no./yr)	0.42 (0.01 to 0.83)	0.79 (0.32 to 1.26)	−0.37 (−0.99 to 0.25)	0.236
SBP (mmHg)	−9.29 (−13.27 to −5.30)	−6.04 (−10.58 to −1.51)	−3.24 (−9.30 to 2.81)	0.284
DBP (mmHg)	−7.30 (−10.36 to −4.23)	−6.44 (−9.93 to −2.95)	−0.86 (−5.52 to 3.80)	0.711
FPG (mmol/L)	−0.30 (−0.50 to −0.10)	−0.07 (−0.29 to 0.15)	−0.23 (−0.53 to 0.07)	0.131
PPG (mmol/L)	−2.12 (−2.82 to −1.41)	−0.86 (−1.65 to −0.08)	−1.26 (−2.31 to −0.20)	0.021
FINS (mU/L)	−7.33 (−12.14 to −2.51)	−7.37 (−12.57 to −2.17)	0.04 (−7.05 to 7.13)	0.990
PINS (mU/L)	−44.11 (−89.04 to 0.82)	−23.37 (−75.98 to 29.25)	−20.74 (−91.08 to 49.60)	0.552
HbA1c (%)	−0.28 (−0.41 to −0.15)	−0.06 (−0.20 to 0.09)	−0.23 (−0.42 to −0.03)	0.027
HOMA-IR	−1.84 (−2.95 to −0.74)	−1.63 (−2.83 to −0.44)	−0.21 (−1.84 to 1.42)	0.794
ALT (U/L)	−16.61 (−21.49 to −11.73)	−14.09 (−19.24 to −8.94)	−2.52 (−9.74 to 4.70)	0.484
AST (U/L)	−6.38 (−8.14 to −4.62)	−6.54 (−8.46 to −4.62)	0.16 (−2.52 to 2.84)	0.904
TC (mmol/L)	−0.62 (−0.83 to −0.41)	−0.52 (−0.75 to −0.28)	−0.11 (−0.42 to 0.21)	0.509
TG (mmol/L)	−0.59 (−0.73 to −0.45)	−0.46 (−0.61 to −0.31)	−0.13 (−0.34 to 0.08)	0.205
LDL-c (mmol/L)	−0.37 (−0.54 to −0.21)	−0.38 (−0.56 to −0.21)	0.01 (−0.23 to 0.25)	0.949
HDL-c (mmol/L)	−0.12 (−0.21 to −0.04)	−0.06 (−0.14 to 0.03)	−0.07 (−0.19 to 0.05)	0.259
Cr (umol/L)	−0.29 (−2.22 to 1.63)	1.49 (−0.54 to 3.53)	−1.78 (−4.62 to 1.05)	0.209
SUA (umol/L)	−24.27 (−53.04 to 4.50)	−44.73 (−76.75 to −12.70)	20.46 (−22.91 to 63.82)	0.345
LH (IU/L)	−0.56 (−4.66 to 3.54)	3.33 (−1.10 to 7.75)	−3.89 (−9.98to 2.21)	0.205
FSH (IU/L)	−0.56 (−1.50 to 0.39)	0.26 (−0.74 to 1.26)	−0.82 (−2.19 to 0.56)	0.237
PRL (mIU/L)	57.91 (−27.24 to 143.06)	7.76 (−85.04 to 100.55)	50.15 (−75.82 to 176.13)	0.423
TT (nmol/L)	−0.06 (−0.26 to 0.14)	−0.22 (−0.43 to −0.01)	0.16 (−0.13 to 0.45)	0.274
FT (pg/mL)	−0.04 (−0.57 to 0.48)	−0.35 (−0.93 to 0.22)	0.31 (−0.48 to 1.10)	0.427
AD (ng/mL)	0.04 (−0.63 to 0.71)	−0.62 (−1.23 to −0.02)	0.67 (−0.24 to 1.57)	0.143
DHEAS (ug/dl)	4.75 (−24.39 to 33.89)	18.02 (−13.02 to 49.07)	−13.27 (−56.05 to 29.51)	0.531
SHBG (nmol/L)	−1.60 (−9.14 to 5.93)	1.52 (−6.01 to 9.06)	−3.13 (−13.96 to 7.70)	0.557
FAI	0.03 (0.01 to 0.04)	0.04 (0.02 to 0.05)	−0.01 (−0.03 to 0.01)	0.351
CAP (dB/m)	−12.46 (−30.56 to 5.64)	−22.35 (−43.91 to −0.79)	9.89 (−18.32 to 38.10)	0.478
LSM (kPa)	−0.83 (−1.53 to −0.13)	−0.35 (−1.18 to 0.48)	−0.49 (−1.57 to 0.60)	0.368
Total body fat (%)	−0.09 (−1.21 to 1.04)	0.09 (−1.15 to 1.33)	−0.18 (−1.86 to 1.51)	0.833
Total body lean (%)	−0.20 (−1.33 to 0.92)	−0.36 (−1.63 to 0.91)	0.16 (−1.55 to 1.87)	0.852
Total fat mass (kg)	−2.11 (−2.98 to −1.23)	−1.95 (−2.93 to −0.96)	−0.16 (−1.48 to 1.17)	0.810
Total lean mass (kg)	−3.14 (−3.98 to −2.31)	−2.77 (−3.71 to −1.82)	−0.38 (−1.64 to 0.88)	0.548
SAT mass (kg)	−0.25 (−0.35 to −0.14)	−0.17 (−0.29 to −0.06)	−0.07 (−0.23 to 0.08)	0.351

Mean differences between the two treatment groups were presented as least-squares mean change with 95% CI. AD, androstenedione; ALT, alanine aminotransferase; AST, aspartate aminotransferase; BMI, body mass index; CAP, controlled attenuation parameter; Cr, creatinine; CRD, calorie-restricted diet; DBP, diastolic blood pressure; DHEAS, dehydroepiandrosterone sulfate; FAI, free androgen index; FINS, fasting insulin; FPG, fasting plasma glucose; FSH, follicle-stimulating hormone; FT, free testosterone; HbA1c, glycosylated hemoglobin A1c; HDL-c, high-density lipoprotein cholesterol; HOMA-IR, homeostasis model assessment of insulin resistance; LDL-c, low-density lipoprotein cholesterol; LH, luteinizing hormone; LS, least-squares; LSM, liver stiffness measurement; PCOS, polycystic ovary syndrome; PINS, postprandial insulin; PPG, postprandial plasma glucose; PRL, pituitary prolactin; SAT, subcutaneous adipose tissue; SBP, systolic blood pressure; SHBG, sex hormone-binding globulin; SUA, serum uric acid; TC, total Cholesterol; TG, triglyceride; TT, total testosterone; WC, waist circumference.

**Table 3:** Adverse events and safety data during the treatment in two groups.

Event	Dulaglutide Combined with CRD Therapy (*n* = 35)	CRD Therapy (*n* = 33)
	No. of Participants (%)	No. of Participants (%)
Adverse events-related discontinuation	2 (5.71)	0 (0)
Patients with ≥1 GI TEAE	13 (37.14)	0 (0)
Nausea	8 (22.86)	0 (0)
Vomiting	7 (20.00)	0 (0)
Diarrhea	0 (0)	0 (0)
Constipation	4 (11.43)	0 (0)
Loss of appetite	4 (11.43)	0 (0)
Abdominal distension	2 (5.71)	0 (0)
Abdominal pain	1 (2.86)	0 (0)
Eructation	1 (2.86)	0 (0)
Sensations of hunger	0(0)	3(9.09)
Hypoglycemia	0 (0)	0 (0)
Dizziness	3 (8.57)	1 (3.03)
Injection site reaction	0 (0)	0 (0)
Upper respiratory tract infection	0 (0)	0 (0)
Headache	1 (2.86)	0 (0)
Nasopharyngitis	0 (0)	0 (0)

CRD, calorie-restricted diet; GI, gastrointestinal; TEAE treatment-emergent adverse event.

## Data Availability

All data generated or analyzed during this study are available from the corresponding authors on reasonable request.

## Supplementary Materials

The following supporting information can be downloaded at: https://www.mdpi.com/article/10.3390/nu15030556/s1, Figure S1: Adherence to prescribed diets in patients who reached the target weight loss within six months during the treatment time. The percentage of adherence to prescribed diets was calculated as adherence/intervention days.
