# Supplementary figures and images for: Effects of a Dulaglutide plus Calorie-Restricted Diet versus a Calorie-Restricted Diet on Visceral Fat and Metabolic Profiles in Women with Polycystic Ovary Syndrome: A Randomized Controlled Trial
Source: Nutrients. 2023 Jan 20;15(3):556. doi: 10.3390/nu15030556 (PMC9920202; doi:10.3390/nu15030556)

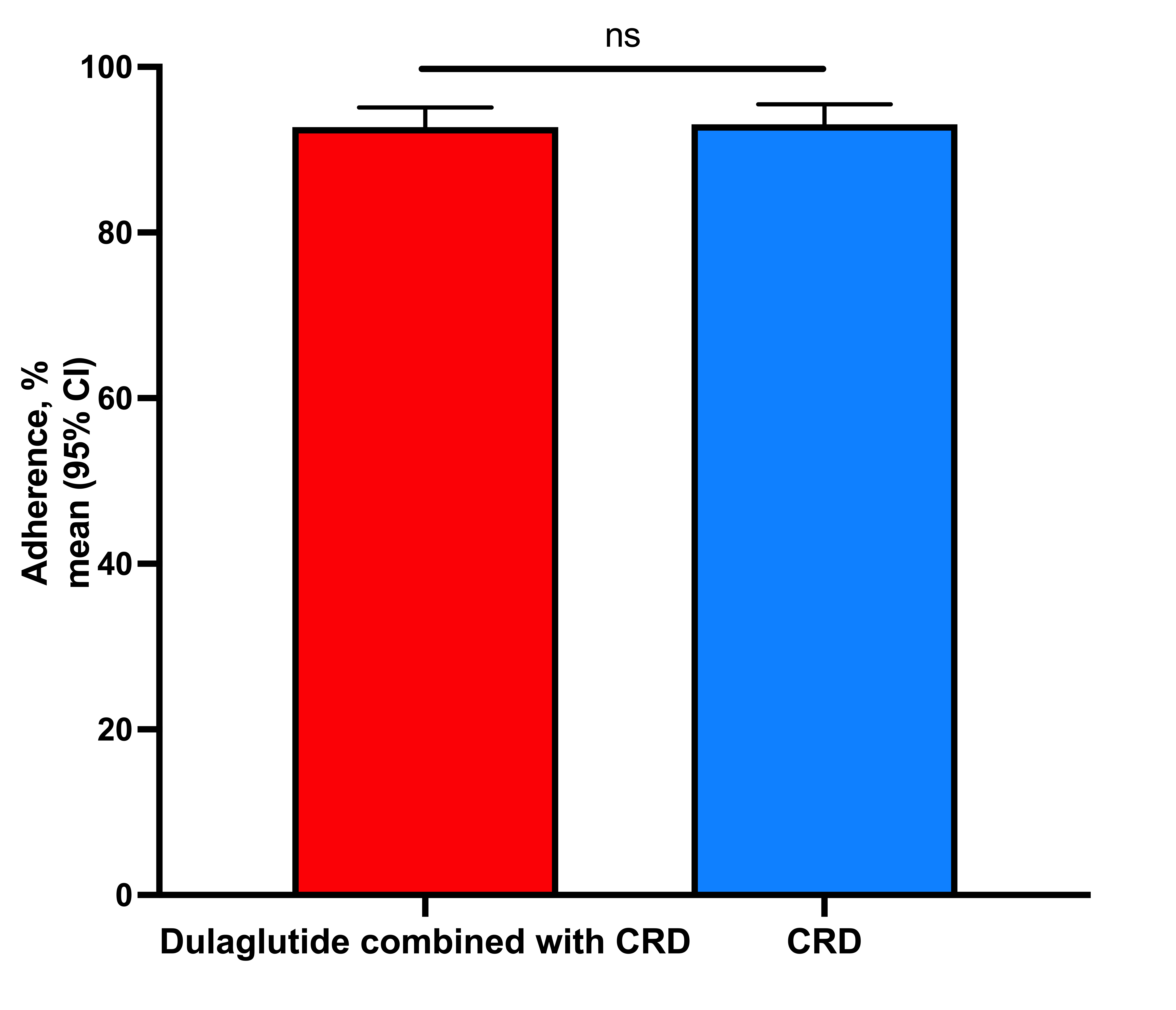

Supplement: Supplementary file 1 [file nutrients-15-00556-s001.zip › Supplementary files/Figure S1.tiff]
